# Strategies to address diabetic kidney disease burden in Mexico: a narrative review by the Mexican College of Nephrologists

**DOI:** 10.3389/fmed.2024.1376115

**Published:** 2024-06-19

**Authors:** Silvia Palomo-Piñón, Jesús Armando Aguilar-Alonso, Jonathan Samuel Chávez-Iñiguez, Felipe Ericel Hernández-Arellanes, Jesús Antonio Mariano-Murga, Juan Carlos Flores-Rodríguez, María Juana Pérez-López, Fabiola Pazos-Pérez, Alejandro Treviño-Becerra, Aurora E. Guillen-Graf, Jesús Manolo Ramos-Gordillo, Pedro Trinidad-Ramos, Neftali Eduardo Antonio-Villa

**Affiliations:** ^1^Vicepresidente del Colegio de Nefrólogos de México AC, Mexico City, Mexico; ^2^Directora General del Registro Nacional de Hipertensión Arterial México (RIHTA) Grupo de Expertos en Hipertensión Arterial México (GREHTA), Mexico City, Mexico; ^3^Servicio de Nefrología, Hospital General de Zona No. 2, Tuxtla Gutiérrez, Mexico; ^4^Servicio de Nefrología, Hospital Civil de Guadalajara Fray Antonio Alcalde, Guadalajara, Mexico; ^5^Departamento de Nefrología, Hospital de Especialidades Dr. Antonio Fraga Mouret, Centro Médico Nacional La Raza, Instituto Mexicano del Seguro Social, Mexico City, Mexico; ^6^Clínica de Diálisis Peritoneal, Hospital General de Zona No. 33, Bahía de Banderas, Mexico; ^7^Departamento de Nefrologia y Trasplante, Hospital General Regional No. 1, Cuernavaca, Mexico; ^8^Nefrología, UMAE Hospital de Especialidades Dr. Bernardo Sepúlveda Gutiérrez, Centro Medico Siglo XXI, Mexico City, Mexico; ^9^Academia Nacional de Medicina, Mexico City, Mexico; ^10^Centro de Diagnóstico Ángeles, Mexico City, Mexico; ^11^Nefrólogo Independiente, Mexico City, Mexico; ^12^Departamento de Endocrinología, Instituto Nacional de Cardiología Ignacio Chávez, Mexico City, Mexico

**Keywords:** diabetic kidney disease, Mexico, chronic kidney disease, diabetes, iSLGT2, GLP-1

## Abstract

Chronic kidney disease (CKD) is a growing global public health challenge worldwide. In Mexico, CKD prevalence is alarmingly high and remains a leading cause of morbidity and mortality. Diabetic kidney disease (DKD), a severe complication of diabetes, is a leading determinant of CKD. The escalating diabetes prevalence and the complex regional landscape in Mexico underscore the pressing need for tailored strategies to reduce the burden of CKD. This narrative review, endorsed by the Mexican College of Nephrologists, aims to provide a brief overview and specific strategies for healthcare providers regarding preventing, screening, and treating CKD in patients living with diabetes in all care settings. The key topics covered in this review include the main cardiometabolic contributors of DKD (overweight/obesity, hyperglycemia, arterial hypertension, and dyslipidemia), the identification of kidney-related damage markers, and the benefit of novel pharmacological approaches based on Sodium-Glucose Co-Transporter-2 Inhibitors (SGLT2i) and Glucagon-Like Peptide-1 Receptor Agonists (GLP-1 RA). We also address the potential use of novel therapies based on Mineralocorticoid Receptor Antagonists (MRAs) and their future implications. Emphasizing the importance of multidisciplinary treatment, this narrative review aims to promote strategies that may be useful to alleviate the burden of DKD and its associated complications. It underscores the critical role of healthcare providers and advocates for collaborative efforts to enhance the quality of life for millions of patients affected by DKD.

## Introduction

**Figure 1:**
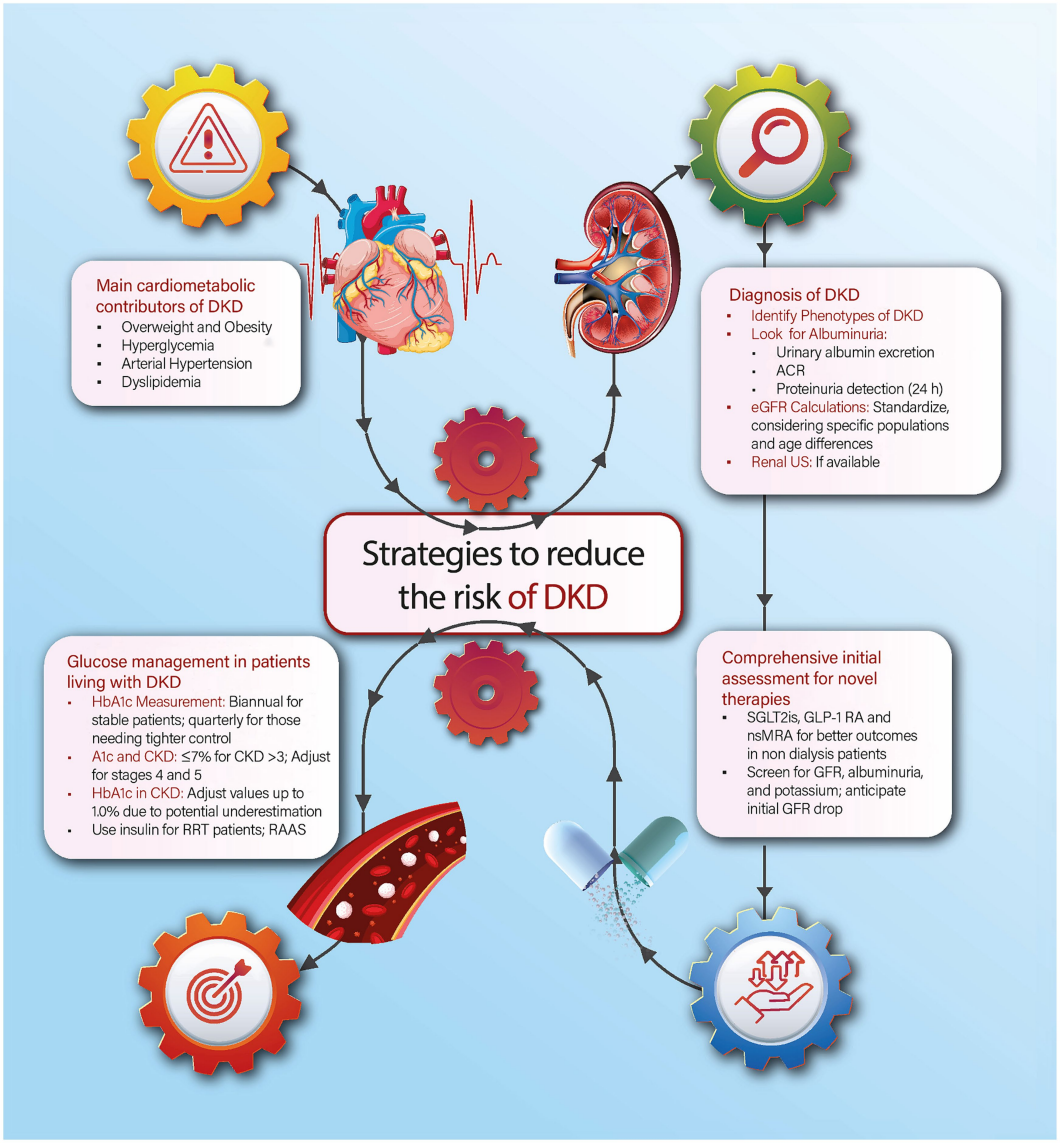
Summary of four strategies to reduce the burden of CKD in patients living with diabetes in the context of Mexico. A1c, Glycated hemoglobin; ACR, Albumin-to-Creatinine Ratio; CKD: Chronic Kidney Disease; DKD, Diabetic Kidney Disease; eGFR, Estimated Glomerular Filtration Rate; GLP-1 RA, Glucagon-Like Peptide-1 Receptor Agonists; nsMRA, Non-steroidal Mineralocorticoid Receptor Antagonists; RAAS, Renin-Angiotensin-Aldosterone System, RRT, Renal Replacement Therapy; SGLT2i, Sodium-Glucose Co-Transporter-2 Inhibitors; US, Ultrasound.

Chronic kidney disease (CKD) is a major public health problem due to its high global prevalence, affecting an estimated 843.6 million adults worldwide (1). In Mexico, it has been estimated that approximately 14.5 million people live with CKD, making it one of the top 10 causes of death, along with diabetes and cardiovascular disease (CVD) (2). Diabetes-induced CKD, also known as diabetic kidney disease (DKD), is a main contributor to CKD, which results from prolonged elevated blood sugar levels and can eventually require dialysis or renal transplantation. The main contributor to DKD is the rising incidence of diabetes (3). According to the International Diabetes Federation (IDF), they estimate that about 536.6 million adults worldwide have diabetes, representing 10.5% of the world’s adult population, and they projected that the burden of diabetes will increase to 783.2 million by 2045. Mexico ranks among the countries with the highest prevalence of diabetes, reaching an astonishing 16.9% in 2022, which results in 14.1 million adults living with this disease (4, 5). Consequently, diabetes is a major contributor to the macro-and-microvascular complications seen in CKD, leading to high mortality rates and significant deterioration in quality of life among these patients (6). However, diabetes does not uniformly result in renal damage, as only one in four patients living with diabetes will develop CKD. Therefore, it is crucial to understand and address the heterogenicity of diabetes and its relationship with CKD to apply clinical strategies and create public health policies to reduce the burden of both diseases in Mexico. This narrative review aims to provide a general overview of the main contributors to CKD in patients living with diabetes and specific strategies for healthcare providers regarding prevention, screening, and treatment. We scope our review on four key areas, as illustrated in Figure 1, including the prevention of cardiometabolic contributors, the identification of kidney-related damage markers, and the benefits of using novel pharmacological approaches. The strategies outlined in this review are designed to enhance the understanding and management of CKD in patients living with diabetes among healthcare providers in all care settings in Mexico.

### Section 1: cardiometabolic contributors to the burden of diabetic kidney disease

Addressing the contributors of DKD’s development can mitigate its burden. Central to this discussion is the multifaceted aspects of cardiometabolic diseases, which are highly prevalent in our country and act as precursors and exacerbators of diabetes, cardiovascular complications, and CKD. In this section, we will discuss the main cardiometabolic contributors to diabetic kidney disease in the context of Mexico.

### Question 1.1 – what are the main cardiometabolic contributors of DKD?

#### Overweight and obesity

The complex interplay between obesity and diabetes leads to overall kidney damage. This damage primarily emerges from neurohormonal shifts in the cardiovascular system, characterized by increased sympathetic activity, accelerating heart rate, and spiking blood pressure levels (7). Central to this is the role of angiotensin II, synthesized in adipose tissue, which interfaces with diverse pathways to induce endothelial dysfunction, inflammation, and fibroblast proliferation (8, 9). Another exacerbator of obesity is related to the growth in the body surface, which often outpaces renal mass expansion, facilitating conditions like hyperfiltration within the glomerular filtration barrier focal and segmental glomerulosclerosis, and glomerulomegaly. Consequently, obesity is considered to be a main risk factor for diabetes and its progression to DKD (10–12).

#### Hyperglycemia

Hyperglycemia, or elevated blood sugar levels, is a primary factor in kidney damage. Several theories have been proposed to explain the relationship between high glucose levels and renal impairment. The main ones include sorbitol formation, oxidative stress escalation, protein kinase C activation, and hexosamine pathway activation (13, 14). However, the most widely accepted theory centers around advanced glycation end products (AGEs). AGEs are complex compounds that form when the residuals of the reduction of sugars are bound to proteins. AGEs can damage cells in several ways. First, they can generate reactive oxygen species (ROS), which can damage DNA. Second, AGEs can activate transcription factors that can produce inflammatory cytokines (15, 16). Third, they can bind to growth factors, such as transforming growth factor β (TGF-β), which can promote fibrosis, and the vascular endothelial growth factor (VEGF), an influential angiogenic mediator (17). In the next sections, we will mention the role of managing blood glucose and how it is an essential factor in reducing the risk of progression to DKD.

#### Arterial hypertension

Arterial hypertension in patients living with diabetes has unique physiopathological features (18–20). These patients tend to experience higher elevations in systolic blood pressure, wider pulse pressures, increased blood pressure variability, heightened salt sensitivity, a tendency toward hyperkalemia, and orthostatic hypotension, among others. These processes are combined with specific CKD-related mechanisms, such as the activation of the renin-angiotensin-aldosterone system (RAAS), stress on the renal microvasculature and the endoplasmic reticulum, mitochondrial dysfunction, and apoptosis, which ultimately promote endothelial cell damage, thickening of the glomerular basement membrane, podocyte effacement, and pedicel fusion (21). Therefore, blood pressure targets are one of the main protective factors to the onset of DKD. In this context, patients’ optimal blood pressure targets are a topic of ongoing debate. Some guidelines and studies recommend aiming for a blood pressure below 140/90 mmHg, while others advocate for even lower targets, such as 130/80 mmHg or a systolic pressure of less than 120 mmHg (22, 23). In terms of pharmacological treatment, medications that have been shown to be effective in lowering blood pressure in the context of DKD include angiotensin-converting enzyme inhibitors (ACEIs) and angiotensin II receptor blockers (ARBs) (23–25). Based on individual patient conditions, diuretics or calcium channel blockers may serve as second-line treatments, with spironolactone positioned as a potential fourth-line option (26, 27). Some clinical trials have highlighted the potential benefit of using chlorthalidone as an alternative to spironolactone for people with refractory hypertension and decreased glomerular filtration rate (28). In patients with absolute or relative contraindications to use ARBs or ACEIs, non-dihydropyridine Calcium Channel Blockers, whose beneficial effect on proteinuria is documented in clinical studies, may be considered (29).

#### Dyslipidemia

Dyslipidemia is highly prevalent in Mexico. In national surveys, hypoalphalipoproteinemia is the most frequent dyslipidemia in Mexico, followed by hypertriglyceridemia (30). The co-existence of dyslipidemia and diabetes is also extremely high. In some studies from other countries, rates have been reported up to 85.3%, with a higher prevalence in females and the elderly (31). The pathophysiological mechanism of kidney damage due to dyslipidemia is linked with lipotoxicity, which generates an exacerbated inflammatory process. Free fatty acids (FFAs) are triacylglycerols stored in adipose tissue, primarily as abdominal fat. When abdominal fat increases, there is a rise in blood FFAs, which exceeds the maximum lipid storage capacity of adipose tissue, releasing lipid mediators that cause insulin resistance and accumulation of fatty acids in other tissues, such as the heart, blood vessels, kidneys, liver, and pancreas. This leads to an increase in the expression of inflammatory cytokines, oxidative stress, lipid peroxidation, and vesicular transport dysfunction, causing endoplasmic reticulum and lysosomal stress. All these alterations promote atherogenesis in renal vasculature and the progression of kidney damage (32). Moreover, it has also been reported that dyslipidemia increases the values of oxidized low-density lipoprotein cholesterol in plasma and renal parenchyma, which are cytotoxic to kidney cells, inducing apoptosis of tubular cells (33). Currently, the term “fatty kidney” has been coined. This condition is characterized by the ectopic accumulation of lipids in kidney tissue, causing lesions at the level of podocytes, proximal tubule epithelial cells, and tubulointerstitial tissue (34).

### Section 2: phenotypes of DKD and their risk for kidney damage, cardiovascular morbidity, and mortality

The hallmark of DKD is characterized by an elevated level of albumin in urine, either >300 mg/24 h or an albumin/creatinine ratio > 300 mg/g of creatinine. To accurately diagnose DKD, these levels should be confirmed in at least two out of three assessments and should be distinct from other kidney etiologies (35, 36). Therefore, screening for albuminuria has become the main indicator of kidney damage in diabetes and its related complications. Nevertheless, the conventional presentation of DKD, which encompasses high albuminuria and a decreasing glomerular filtration rate (GFR), is found in only 30–40% of patients living with diabetes (35). In this section, we will discuss the strategies for evaluating microalbuminuria and GFR and shed light on the diverse DKD phenotypes.

### Question 2.1 – what are the recommended tests to detect albuminuria?

In clinical practice, the primary markers for kidney function are albuminuria and GFR (37). Albuminuria has been demonstrated to correlate with an increased risk of GFR decline, CKD progression, and a risk factor for CVD-related morbidity and mortality. Therefore, in patients living with diabetes, albuminuria onset serves as an early marker for DKD. Table 1 presents the advantages and disadvantages of three tests used to measure albuminuria in the context of low-and middle-income countries based on recent recommendations (38):

1. Urinary Albumin Excretion: This can be measured either with a random urine sample or through monitoring albumin excretion over a 24-h urine collection.
2. Albumin/Creatinine Ratio (ACR): The albumin/creatinine ratio (ACR) has emerged as a standardized way to measure albuminuria (39). Based on ACR, it has been suggested that a level > 300 mg/g is a strong risk factor for DKD progression (40). This can be determined from a random or from a 24-h urine collection.
3. Proteinuria Detection: Measured using a reagent strip.

**Table 1:** Methods for the detection of kidney damage related to DKD.

	Determination of albuminuria in 24-h urine	Albumin/creatinine ratio in a random urine sample	Dipstick proteinuria
Advantages	► Specific and precise quantification at low concentrations ► Quantitative results in the clinically relevant range.	►Preferred by guidelines. ► Specific and precise quantification at low concentrations ► Quantitative results in the clinically relevant range. ►More sensitive to changes in glomerular permeability	►Convenient ►Lower cost
Disadvantages	► Expensive and time consuming and adds little to prediction. ►Repeat testing is required, with 2 of 3 abnormal measurements within a 3–6-month period before a patient is considered to have albuminuria.	►Repeat testing is required, with 2 of 3 abnormal measurements within a 3–6-month period before a patient is considered to have albuminuria	►Only detects albuminuria ►Measurement of albumin alone without simultaneous measurement of urine creatinine is susceptible to false-negative and false-positive results

According to the Kidney Disease: Improving Global Outcomes (KDIGO) guidelines, it is suggested that determining albumin excretion over a 24-h urine collection should be the initial method for screening kidney damage in patients with diabetes (41). If the clinical circumstances make this test unfeasible, albumin excretion from a random urine sample could be a reliable alternative for evaluating kidney disease in diabetes. Both methods offer the most precise evaluations for diabetes-related kidney damage in clinical scenarios. Additionally, the ACR is more accurate than the 24-h urine collection. However, both the 24-h urine collection and the ACR have been reported to be impractical and challenging in primary care settings (41, 42).

In low-to-middle-income regions, such as Mexico, the widespread availability of reagent strip tests for urinary protein has also been pinpointed as an alternative to measure albuminuria. Nevertheless, previous evidence has shown its limited effectiveness within clinical contexts. Some studies have highlighted its diminished sensitivity and specificity when identifying albuminuria, given its semi-quantitative method that can detect only 30–40% of urinary proteins (41, 43). Hence, this test is not recommended to evaluate albuminuria in primary or routine clinical screening or follow-up visits.

### Question 2.2 – does proteinuria hold the same prognostic value as albuminuria for renal function and the development of cardiovascular complications?

When assessing patients with DKD, it is essential to consider albuminuria rather than proteinuria as a marker of progression of the disease. However, both metrics are essential when evaluating individuals with non-diabetic CKD and for screening kidney damage in the general population (44). Urinary albumin is the main protein lost in the urine, and its measurement provides a more accurate assessment of glomerular permeability changes than evaluating total proteins in the urine (45, 46). This is also supported by the KDIGO guidelines, which recommend using albuminuria for CKD staging and monitoring (41). However, estimating albuminuria can be limited by higher costs and limited availability in some regions. A summary of the main comparisons between the use of proteinuria and albuminuria is presented in Table 2.

**Table 2:** Clinical comparison between the use of proteinuria and albuminuria as markers for the as markers of progression of the DKD.

	Proteinuria	Albuminuria
Type of damage	Tubulointerstitial damage	Glomerular damage
Higher sensitivity to low levels of urinary proteins	⬆	⬆ ⬆ ⬆
Cost	⬆	⬆ ⬆ ⬆
Analytical test performance	⬆	⬆ ⬆ ⬆
Biomarker for CKD, cardiovascular disease, and mortality in diabetics	⬆	⬆ ⬆ ⬆
Biomarker for CKD, cardiovascular disease, and mortality in non-diabetics	⬆ ⬆ ⬆	⬆
Early diagnosis of diabetic kidney disease	–	⬆ ⬆
Alternative endpoint for the decline in GFR in nephrotic syndrome	⬆ ⬆	–

Adapted from: Guh JY. Proteinuria versus albuminuria in chronic kidney disease. Nephrology. 2010 Jun; 15: 53–6; CKD, chronic kidney disease, CV, cardiovascular, GFR, glomerular filtration rate.

### Question 2.3 – what is the ideal formula to estimate the GFR based on specific populations?

#### Adult population without dialysis requirement

Patients with detected DKD experience an average annual decrease in GFR of 10 to 20 mL/min/1.73m^2,^ which can lead to End-Stage Renal Disease (ESRD) within 7–8 years (47, 48). Therefore, GFR is an important marker in determining the progression of DKD and predicting the onset of ESRD. In clinical practice, the most commonly used equations to determine the estimated GFR (eGFR) in patients aged 18 to 70 years are the CKD Epidemiology Collaboration (CKD-EPI) and the Modification of Diet in Renal Disease by Isotope-Dilution Mass Spectrometry (MDRD-IDMS) (49). MDRD-IDMS and CKD-EPI equations effectively estimate GFR levels below 60 mL/min/1.73m^2^, but CKD-EPI is superior for rates between 60 and 120 mL/min/1.73m^2^ (50). However, efforts to standardize both formulas for specific populations have yielded varied results. A 2021 systematic review from Latin American countries found no significant differences between the CKD-EPI and MDRD-4-IDMS equations. However, most of these studies focused on Brazilian populations, which limits regional extrapolation (49).

#### Older adults living with CKD

A specific situation arises when managing older adults living with DKD. Two new equations based on serum creatinine and cystatin C have emerged for this specific population. The equation using serum creatinine is called BIS-1, while the one that includes CysC is called BIS-2. Both equations were more accurate than MDRD-IDMS and CKD-EPI within older adults (51, 52). In 2016, a novel equation emerged specifically for patients over 70 years: the FAS (Full Age Spectrum). The FAS equation was better than CKD-EPI in classifying CKD stages, especially when GFR ranged from 15 to 60 mL/min/1.73 m2 (53), and it provided similar results to the BIS-1 equation. Therefore, it has been suggested that using the FAS and BIS formulas is a better approach to measuring the eGFR in older adults.

#### Cystatin C-based formulas

It has been reported that serum Cystatin C (CysC) is a more accurate indicator of kidney function compared to creatinine (54). As CysC measurements become more widely available in clinical labs, it may be beneficial to consider using CysC-based equations for more accurate estimations whenever possible, particularly in cases where creatinine-based equations might produce inaccurate results due to fluctuating creatinine concentrations, muscle mass/diet changes, or when GFR is between 45 and 59 mL/min/1.73 m^2^ without other damage markers (55). However, the current KDIGO guidelines advise against using CysC routinely due to its potential cost and the need for specialized equipment (47, 48). Therefore, its use may be potentially reserved in a context with high resources.

#### Pediatric patients living with CKD

The pediatric population presents a challenging scenario when it comes to measuring the eGFR, as several equations have been proposed. Among them, the Schwartz equation is the most recommended formula. However, it tends to overestimate the eGFR since it is based on the Jaffé method for measuring creatinine (56). To achieve better results, it has been suggested that the 2012 Schwartz equation is a better approach as it incorporates serum creatinine, cystatin C, blood urea nitrogen, height, and gender (56, 57).

### Question 2.4 – do the different DKD phenotypes confer the same risk of renal and cardiovascular outcomes in patients with diabetes?

Four different DKD phenotypes have been described during the last decade. The classic DKD phenotype is still the most common; however, current evidence suggests that non-classic phenotypes have increased in prevalence, suggesting that 20–40% of DKD belongs to non-classic phenotypes (45).

- *Classic phenotype*: Patients who experience the classic phenotype typically have a high GFR, which suggests that glomerular hyperfiltration is an adaptive response to nephron loss. This is followed by persistent albuminuria, which is a constant, progressive, and ascending finding characterized by a transition from normo (ACR < 30 mg/g) to micro (ACR 30–299 mg/g) and macro-albuminuria (ACR ≥300 mg/g). As albuminuria progresses, there is usually a continuous decline in GFR, leading to advanced CKD stages.
- *Albuminuria regression phenotype*: In some patients, the progression from normal to macro-albuminuria is not linear, as it has been documented that regression of albuminuria can spontaneously occur in up to 51% of the cases (58). Some studies have investigated the association between regression to normo-albuminuria and the risk of renal function outcomes. In two cohorts of patients with type 1 and 2 diabetes, remission to normo from micro-albuminuria or macro-albuminuria over 4 to 5 years was associated with favorable renal outcomes compared with stable normo-albuminuria (59, 60). However, other studies have shown that the remission to normo from micro-albuminuria was not associated with a reduction in the risk of renal and cardiovascular events (61). These contradictory results may be due to differences in the characteristics of the groups, such as the duration of micro-albuminuria before the study and the use of pharmacological interventions. Therefore, the leading causes of regression and its predictive factors are still an area for further research.
- *Rapid GFR decline phenotype*: The rapid GFR decline phenotype is a condition where patients with diabetes experience a fast decline in their GFR over a brief period. While the annual rate of decline in GFR for most patients with diabetes is between −4.0 to −1.5 mL/min/1.73 m^2^ per year, some patients will experience a much more rapid decline. A recent study conducted on 1,955 people living with diabetes found that up to 14% had a rapid decline in GFR (62). Another study conducted on 377 biopsy-proven diabetic nephropathy cases, with or without albuminuria, reported that 61% of patients experienced a rapid decrease in GFR over a 6.9-year follow-up period (63). High GFR, high systolic blood pressure, or albuminuria were found to be common risk factors for rapid GFR decline and, therefore, should be treated as key determinants of developing this condition.
- *Non-albuminuric rapid GFR decline phenotype*: This phenotype is characterized by a decrease in GFR in the absence of proteinuria. Risk factors for this phenotype include female sex, hypertension, smoking, absence of diabetic retinopathy, and use of RAAS inhibitors (64). Furthermore, this phenotype has a discordance between tubulo-interstitial and vascular lesions and global glomerulosclerosis (64). This data supports the possible involvement of nephrosclerosis due to aging and hypertension, interstitial nephritis and fibrosis, and ischemic vascular disease due to atherosclerosis in the progression of this phenotype. Hence, this phenotype has been described as less aggressive than patients with DKD and proteinuria; however, caution and routine care for these patients should be encouraged.

In the context of Mexico, the following recommendations are endorsed to reduce the progression of kidney damage and cardiovascular morbidity:

1. *Mass screening of albuminuria for high-risk populations:* Early detection, especially in patients living with diabetes, can prevent complications and facilitate timely medical intervention. In Table 1, we summarize the main methods for the detection of albuminuria in clinical practice.
2. *Standardization of GFR*: As previously discussed, equations such as CKD-EPI and MDRD-IDMS play a relevant role in estimating GFR in adults. However, the challenge lies in their applicability across various populations, such as older adults, patients with inadequate creatinine clearance, and the pediatric population. We suggest using customized formulas designed for these specific populations to ensure accuracy.
3. *Integration of diagnostic methods into primary care units:* For a tangible impact on early DKD detection and treatment, it is imperative to incorporate these diagnostic approaches into primary care units, where most of the patients living with diabetes are managed. Early diagnosis of DKD enables prompt initiation of clinical and pharmacological treatment.

### Section 3: glucose management in patients living with DKD

In patients living with diabetes, hyperglycemia tops the list of risk factors for the progression of DKD and ESRD. Moreover, the burden of DKD for patients with diabetes goes beyond kidney function by increasing the risk of CVD and all-cause mortality (65). Therefore, effective glucose management in patients living with diabetes is paramount as it stands as the primary preventive measure not only against micro and macrovascular complications but also to reduce the risk of DKD-related complications. This section will focus on current pharmacological strategies to improve hyperglycemia management, highlighting their kidney-related benefits.

### Question 3.1 – what is the recommended HbA1c target for different CKD stages?

HbA1c is a pivotal metric in assessing glycemic control in patients living with diabetes. To achieve appropriate glycemic control in patients with diabetes and CKD, the American Diabetes Association (ADA) and KDIGO recommend biannual HbA1c evaluations for those meeting therapeutic targets. Conversely, for patients willing to have more intensive glycemic control, those not reaching their glycemic goals, or those undergoing therapeutic adjustments, the ADA prompts that HbA1c measurement should be performed every 3 months (66, 67).

#### HbA1c targets in patients with DKD

**Figure 2:**
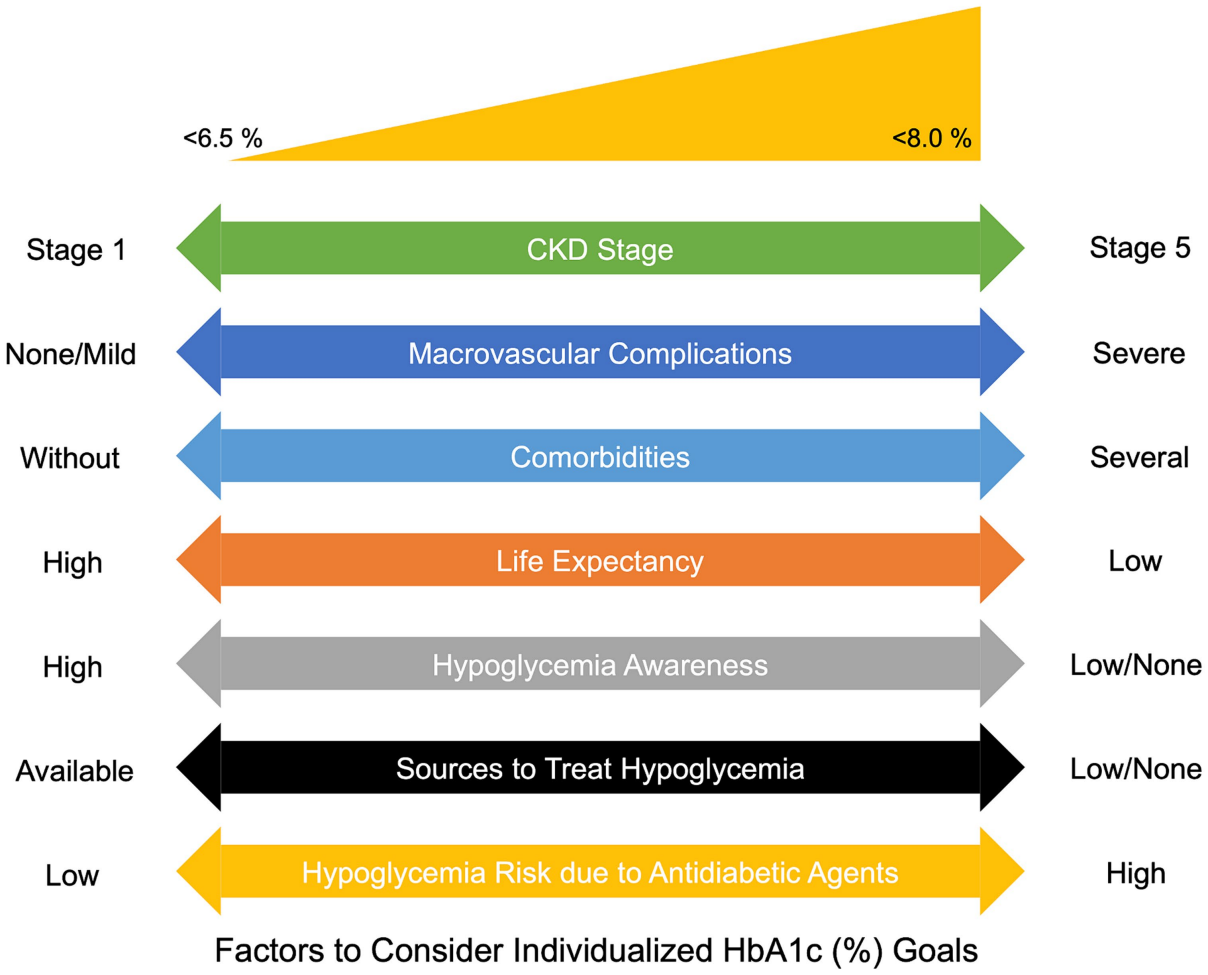
Factors to consider individualized HbA1c goals in patients living with DKD. CKD: Chronic Kidney Disease; HbA1c, Glycated hemoglobin.

Regarding glycemic control, ADA guidelines propose an HbA1c target of ≤7% in patients living with CKD to decrease the risk of CVD complications (68–70). Previous studies suggest that patients living with diabetes with HbA1c levels between 6.5–7.0% experience a reduced risk of micro and macro-albuminuria and the onset of advanced CKD (71). Similarly, other studies have highlighted the benefits of intensive glycemic control in slowing the risk of progression to DKD (72, 73). However, reaching a threshold <6.5% did not result in a significant benefit for the reduction of CVD (74). Overall, both ADA and KDIGO recommend a personalized approach to setting glycemic targets. In Figure 2, we summarize the main factors to consider individualized HbA1c goals in the context of DKD. These strategies need a collaborative effort between patients and healthcare professionals, ensuring that individual risk factors and the potential benefits of glycemic control are adequately considered. Although a general agreement leans toward maintaining HbA1c levels ≤7.0%, dropping toward a Hba1c <6.5% might inadvertently increase hypoglycemia risk. Additionally, the ADA suggests slightly elevated targets (HbA1c <8%) for specific patient groups, especially those with a limited lifespan, severe complications, multiple comorbidities, or a history of hypoglycemic incidents (75).

#### HbA1c in the context of advanced CKD

A challenging scenario in diabetes care emerges when managing glycemic control in patients living with diabetes with advanced stages of CKD, specifically stages 4 & 5 (68, 76). The discussion becomes even more pronounced for patients undergoing renal replacement therapy (RRT). To achieve optimal glucose management within these populations, there are several factors that need to be taken into account. These include the reduction of renal gluconeogenesis due to a decrease in renal tissue volume, slower metabolism and excretion of insulin and oral diabetic medications, which prolongs the half-life of these drugs, and the development of anorexia, decreased food intake, and subsequent weight loss due to the uremic condition (77).

Another important consideration is the reliability of HbA1c as a marker of glycemic control in patients with GFR <30 mL/min/1.73 m^2^ and anemia due to a curtailed accumulation of glycosylation products and hemoglobin carbamylation (78). These spontaneous processes result from the binding of urea metabolism to protein amino groups. Therefore, carbamylation in the context of CKD may artificially increase and fluctuate HbA1c levels (71, 79). In these scenarios, when GFR falls <30 mL/min/1.73 m^2^, HbA1c measurements might be underestimated by 0.5 to 1.0%. Therefore, adjusting observed values of HbA1c within this range could lead to more accurate estimations to evaluate glycemic control in this population. Another potential option is the use of glycated albumin since it’s not influenced by anemia or other CKD-related treatments. Furthermore, it reflects blood glucose status in an average of 2 to 3 weeks (80). Nevertheless, its application may be restricted to its cost and accessibility in several primary care settings.

### Question 3.2 – which hypoglycemic agents have shown benefits on renal and cardiovascular outcomes in CKD before RRT?

The use of sodium-glucose co-transporter-2 inhibitors (SGLT2i) and glucagon-like peptide-1 receptor agonists (GLP-1 RA) has enriched the landscape of diabetes care. These hypoglycemic agents have shown cardiovascular and renal benefits not only for patients living with diabetes but also for those living with CKD (81). This section will discuss the mechanism of action, clinical benefits, and potential side effects of these medications for patients with diabetes and CKD.

#### SGLT2 inhibitors

SGLT2i act by blocking glucose reabsorption in the proximal convoluted tubule via the SGLT-2 co-transporter, thus leading to glycosuria. By inhibiting the SGLT-2 cotransporter, these drugs also reduce sodium reabsorption, which is typically elevated in patients with diabetes due to excessive tubular glucose loads, thus resulting in natriuresis and decreased intravascular volume and blood pressure. Additionally, natriuresis increases sodium supply to the macula densa by normalizing the tubuloglomerular feedback (82). Therefore, it has been described that SGLT2i reduces intraglomerular pressure by constricting abnormally dilated afferent arterioles, thus mitigating glomerular hyperfiltration and delaying the progression of kidney disease. Other pleiotropic effects that explain the renal benefits of SGLT2i include blood pressure reduction, weight loss, and decreased serum uric acid levels. Additionally, SGLT2i possesses anti-inflammatory and antifibrotic properties, which may counteract renal hypoxia and thus reduce systemic inflammation (83). Nevertheless, their effect depends on renal function, which may lead to pharmacodynamic changes in individuals with reduced kidney function (84). Thus, initiating SGLT2i in patients with GFR <20 mL/min/1.73 m^2^ may not be effective and may even increase the risk for adverse events (85).

#### SGLT2 inhibitors in the context of CKD

SGLT2i have shown a clear benefit in reducing CKD progression and preventing ESRD onset (82). Although the absolute risk reduction is higher among cases with severe albuminuria, the relative risk reduction of renal failure is similar among patients with and without this condition. Several clinical trials have found that canagliflozin, dapagliflozin, and empagliflozin can reduce the risk of ESRD, serum creatinine doubling, heart failure hospitalization, CVD death, and all-cause mortality in patients with diabetes and certain levels of GFR and albuminuria/creatinine ratios (86–88). Ertugliflozin has also been studied and was found to have a slower decline in eGFR and a decrease in urine ACR, thus proving efficacy and safety among patients living with diabetes (89, 90). Other SGLT2i, such as ipragliflozin and luseogliflozin, have been studied in smaller trials with diverse renal outcomes, thus leading to inconclusive results for clinical practice (91, 92).

Side effects of SGLT2i include genital fungal infections, for which approximately 10% of women and 1–2% of uncircumcised men had reported. Other studies have reported an increased rate of orthostatic hypotension, especially when combined with diuretics, and an increase in the risk of euglycemic diabetic ketoacidosis (93). An increased risk of below-the-knee amputations was observed with canagliflozin, whil this was not reported for empagliflozin, dapagliflozin, or ertugliflozin as an adverse outcome in their respective trials (94).

#### GLP-1 receptor agonists

GLP-1 RA have emerged as novel and efficient hypoglycemic agents with significant benefits on cardiovascular and renal outcomes (95). Therefore, for patients living with diabetes and CKD who have not attained glycemic control despite initial glucose-lowering therapy or SGLT2i use, GLP-1 RA represent an attractive alternative. This option not only enhances glycemic control but also offers added renal advantages. GLP-1 RA promote a glucose-dependent insulin response by targeting GLP-1 receptors. These are found in the gastrointestinal tract and other tissues, including glomerular endothelial cells. When administered subcutaneously, GLP-1 RA amplify glucose-dependent insulin secretion, diminish glucagon release, decelerate gastric emptying, and suppress appetite. This multifaceted action can lead to substantial weight loss. Furthermore, GLP-1 RA mitigate albuminuria and mesangial expansion by inhibiting TGF-β signaling (96).

#### GLP-1 receptor agonists in the context of CKD

Several clinical trials have assessed the long-term impacts of liraglutide on the composite of diverse clinical outcomes, including improved GFR and decreased risk for CKD onset, kidney disease-related mortality, and CVD mortality. Furthermore, patients treated with liraglutide experienced fewer adverse outcomes compared with conventional treatment (97–99). However, no discernible cardiovascular or CKD benefits were observed with the extended release of either exenatide or lixisenatide (100, 101).

#### Comparison of SGLT2i and GLP-1 RA for albuminuria treatment

A series of systematic reviews focusing on these two drug groups and their effects on ACR in patients living with diabetes concluded that over extended periods, both canagliflozin and empagliflozin decreased ACR by 19–22% compared with control groups, while GLP-1 RA led to a 17–33% reduction in ACR (102). In patients without diabetes, two multicentric clinical trials found that SGLT2i consistently lowers ACR compared to control groups, irrespective of initial albuminuria status (103). In contrast, GLP-1 RA exhibited diverse impacts on ACR reduction based on initial albuminuria status. Compared to placebo, liraglutide led to ACR reductions of 14, 24, and 13% in patients with normo, micro, and macroalbuminuria, respectively (102).

Based on the research and clinical insights related to glucose management in patients living with DKD, we present the following suggestions tailored for the Mexican context:

1. *Perform biannual or trimonthly A1c measurement*: Both ADA and KDIGO guidelines suggest that A1C measurements should be conducted biannually for patients meeting their therapeutic targets. However, for those aiming for tighter glycemic control, those not achieving their glycemic objectives, or those undergoing intensive treatment modifications, the ADA advises quarterly (every 3 months) A1c assessments.
2. *A1c goals should be adapted for CKD stages*: For patients with CKD stages >3, an A1c target of ≤7% is advised. However, for patients with CKD stages 4 and 5, A1c targets should be tailored based on comorbidities and the risk of hypoglycemic episodes.
3. *Acknowledge the* var*iability of HbA1c in the context of CKD*: HbA1c levels can be underestimated in the context of CKD. Consequently, adjusting observed HbA1c values by a factor of 0.5 to 1.0% might provide a more reliable representation of glycemic status.
4. *Consider dose adjustment and novel therapies for patients with diabetes and CKD*: While insulin remains the go-to treatment for those patients undergoing RRT, hypoglycemic medications should be adjusted based on GRF. Table 3 summarizes the main dosage adjustments based on hypoglycemia families. Furthermore, for those failing to achieve satisfactory glycemic control with conventional therapies, introducing SGLT2i and GLP-1 RA should be considered. These novel agents can enhance glucose regulation and diminish the risk of adverse renal and CVD outcomes.

**Table 3:** Dosage adjustment for medications for the treatment of diabetes for subjects with chronic kidney disease (CKD) stages 3–5 and renal replacement therapy.

Pharmacological group	Dosage adjustment
Insulin	No dose adjustment
Sulfonylureas	Glipizide: No dose adjustment Glyburide: Avoid its use
Biguanides	Metformin: Suspend treatment with eGFR <30 mL/min/1.73m^2^
Thiazolidinediones	Pioglitazone: No dose adjustment
DPP-4 Inhibitors	Linagliptin: No dose adjustment Sitagliptin: eGFR 30–50 mL/min/1.73m^2^ adjust to 50 mg/day and eGFR <30 mL/min/1.73m^2^ to 25 mg/day
GLP-1 receptor agonists	No dose adjustment required
SGLT-2 inhibitors	Canagliflozin and empagliflozin not recommended with eGFR <30 mL/min/1.73m^2^ Dapagliflozin use not recommended with eGFR <25 mL/min/1.73m^2^

### Section 4: other pharmacological approaches to treat DKD

Patients living with DKD often display neurohormonal hyperactivity, which elevates their risk for adverse CVD and renal outcomes. Pathophysiological pathways in the cardiorenal syndrome are characterized by a sustained inflammatory response and increased RAAS activity. The combined effect of neurohormonal and RASS hyperactivity results in increased water and sodium retention, leading to volume overload (104), and cardiac and renal fibrosis, which is then aggravated with a functional decline (105). Although these pathways are linked and share similar pathophysiological traits, there are different progression patterns in renal function for these patients, such as rapid GFR decline and fluctuating GFR trajectories, which have implications for patient survival (64). This section will discuss new pharmacological methods to treat DKD, considering other pathophysiological pathways involved.

### Question 4.1 – what is the evidence of novel mineralocorticoid receptor antagonists for DKD?

RAAS inhibitors, ARBs, and SGLT2i have consistently been demonstrated to decrease the progression of DKD (106–108). However, around 35% of patients will face an elevated risk of progressing to more severe stages of CKD related to high blood pressure despite using these medications (109). This enduring risk might be attributed to a phenomenon called “aldosterone escape,” which is experienced by almost half of the patients treated with RAAS inhibitors within the first year (110). The “aldosterone escape” is characterized by a significant decrease in the antiproteinuric effects of RAAS despite receiving appropriate pharmacological therapy (110). While aldosterone primarily regulates sodium and potassium balance in the nephron, its secondary effects can induce the expression of inflammation markers (such as SGK1, MCP1, TGF-β1, and IL-6), which overall contribute to hypertrophy and fibrosis related to DKD (111, 112). Furthermore, it has been demonstrated that increased aldosterone levels serve as a marker of renal damage and are associated with requiring RRT and all-cause mortality (113). Similar studies have suggested that even short durations of aldosterone stimulation can induce renal tubular interstitial fibrosis (114, 115).

Over the past 30 years, clinical efforts have been made to mitigate aldosterone’s deleterious effects through mineralocorticoid receptor antagonists (MRAs) primarily to decrease blood pressure levels (111, 116). The combination of MRAs and RAAS inhibitors has demonstrated benefits by decreasing proteinuria levels whil preserving GFR levels (115). Additionally, MRAs have been shown to provide hemodynamic benefits by reducing both systolic and diastolic blood pressure levels by an average of 5.6 and 1.73 mmHg, respectively (117). However, their use has also been associated with an elevated risk of hyperkalemia, which has limited its implementation, particularly in patients living with diabetes and undergoing RRT (118, 119).

### Question 4.2 – what is the clinical evidence for using finerenone to treat DKD?

The challenges with older MRAs led to the development of finerenone, which has a stronger affinity to mineralocorticoid receptors (119). Unlike spironolactone and eplerenone, finerenone does not affect the central nervous system and has decreased side effects compared with traditional MRAs (120). Moreover, it effectively reduces sodium channel activity in the kidney, promoting salt excretion and reducing inflammation and scarring in other cell types (121). In two multicenter clinical trials and combined analyses, finerenone reduced, on average, the systolic blood pressure by 3.8 mmHg, reduced kidney and heart-related damage, and lowered the risk of requiring RRT in the long term (122–125). Moreover, other clinical trials have shown that finerenone offers kidney benefits similar to SGLT2i (126, 127), and only a few patients discontinued treatment due to high potassium levels (126, 128).

**Figure 3:**
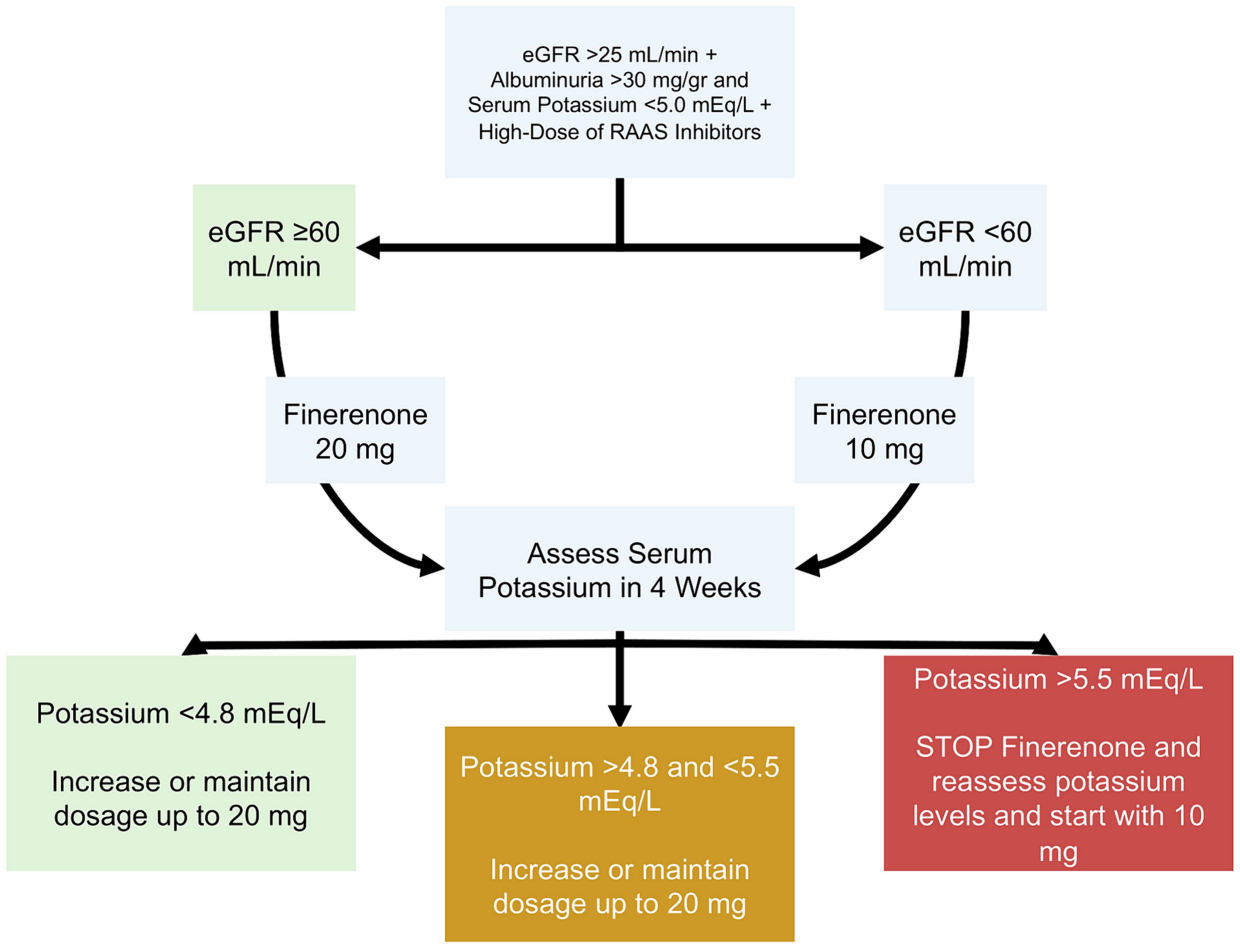
Proposed dosing of finerenone in patients with diabetes. eGFR, Estimated Glomerular Filtration Rate; RAAS, Renin-Angiotensin-Aldosterone System.

In the context of Mexico, the following suggestions are endorsed to consider the complementary use of finerenone to decrease the risk of progression of DKD:

1. *Comprehensive initial assessment*: Clinicians should conduct extensive screening before initiating finerenone, emphasizing the baseline GFR, albuminuria, and serum potassium levels. According to some studies, there is an initial decrease in the GFR, which eventually stabilizes over time (122, 125).
2. *Safety and dosage protocols:* Figure 3 summarizes the recommendations for initiating finerenone. A 10 mg/day dosage is recommended for patients with a baseline potassium level below 5.0 mEq/L. Monitoring and adjusting the dosage based on dynamic potassium levels is important to ensure patient safety. It should be noted that approximately 5% of patients might experience elevated serum potassium levels (129).
3. *Adherence to endorsed guidelines*: When prescribing finerenone to diabetic patients, it’s important to follow ADA and KDIGO guidelines, especially when prescribed with RAAS inhibitors (130).

## Conclusion

In conclusion, our review outlines four strategies to reduce the burden of CKD in patients living with diabetes in the context of Mexico. Early detection of albuminuria, adequate glucose control, and addressing cardiometabolic contributors are the main strategies for reducing the progression of DKD in patients living with diabetes. The use of new treatments based on SGLT2i and GLP-1 RA have shown potential benefits in reducing overall kidney damage and the risk of complications associated with DKD. Additionally, novel therapies based on MRAs are gaining interest due to their promising results, which could be adopted in the future. Implementing these strategies into clinical practice could help improve outcomes and enhance patient survival and quality of life.

## Author contributions

SP-P: Writing – original draft, Writing – review & editing. JA-A: Writing – original draft. JC-I: Writing – original draft. FH-A: Writing – original draft. JM-M: Writing – original draft. JF-R: Writing – review & editing. MP-L: Writing – review & editing. FP-P: Writing – original draft. AT-B: Writing – original draft. AG-G: Writing – original draft. JR-G: Writing – original draft. PT-R: Writing – review & editing. NA-V: Writing – review & editing.
